# Human forebrain organoids reveal connections between valproic acid exposure and autism risk

**DOI:** 10.1038/s41398-022-01898-x

**Published:** 2022-03-29

**Authors:** Qingtuan Meng, Wendiao Zhang, Xuan Wang, Chuan Jiao, Sheng Xu, Chunyu Liu, Beisha Tang, Chao Chen

**Affiliations:** 1 The First Affiliated Hospital, Multi-Omics Research Center for Brain Disorders, Hengyang Medical School, University of South China, 421001 Hengyang, Hunan China; 2 Center for Medical Genetics & Hunan Key Laboratory of Medical Genetics, School of Life Sciences, and Department of Psychiatry, The Second Xiangya Hospital, Central South University, 410008 Changsha, Hunan China; 3 Department of Psychiatry, SUNY Upstate Medical University, Syracuse, NY 13210 USA; 4 Hunan Key Laboratory of Animal Models for Human Diseases, Central South University, 410008 Changsha, Hunan China; 5 Hunan Key Laboratory of Molecular Precision Medicine, Central South University, 410008 Changsha, Hunan China

**Keywords:** Autism spectrum disorders, Stem cells, Molecular neuroscience

## Abstract

Valproic acid (VPA) exposure as an environmental factor that confers risk of autism spectrum disorder (ASD), its functional mechanisms in the human brain remain unclear since relevant studies are currently restricted to two-dimensional cell cultures and animal models. To identify mechanisms by which VPA contribute to ASD risk in human, here we used human forebrain organoids (hFOs), in vitro derived three-dimensional cell cultures that recapitulate key human brain developmental features. We identified that VPA exposure in hFOs affected the expression of genes enriched in neural development, synaptic transmission, oxytocin signaling, calcium, and potassium signaling pathways, which have been implicated in ASD. Genes (e.g., *CAMK4*, *CLCN4*, *DPP10*, *GABRB3*, *KCNB1*, *PRKCB*, *SCN1A*, and *SLC24A2*) that affected by VPA were significantly overlapped with those dysregulated in brains or organoids derived from ASD patients, and known ASD risk genes, as well as genes in ASD risk-associated gene coexpression modules. Single-cell RNA sequencing analysis showed that VPA exposure affected the expression of genes in choroid plexus, excitatory neuron, immature neuron, and medial ganglionic eminence cells annotated in hFOs. Microelectrode array further identified that VPA exposure in hFOs disrupted synaptic transmission. Taken together, this study connects VPA exposure to ASD pathogenesis using hFOs, which is valuable for illuminating the etiology of ASD and screening for potential therapeutic targets.

## Introduction

Autism spectrum disorder (ASD) is a common neurodevelopmental disorder characterized by impaired social communication and interaction, as well as repetitive and stereotypic behaviors [1]. The heritability of ASD reaches 80–90% [2, 3], suggesting the essential roles of genetic factors in ASD etiology. To date, hundreds of genes harboring genetic mutations have been identified as ASD risk genes [4, 5]. In addition to a strong genetic component [3], environmental factors such as maternal infection, toxin or drug exposure are known to confer ASD risk [6]. Pregnancy exposure to valproic acid (VPA), a clinical drug used to treat epilepsy and mood disorders [7], has been found to be associated with increased ASD risk in the offspring [8]. Researchers found that the absolute risk of ASD among VPA-exposed children was 4.15% (95% CI, 2.20–7.81%), and was 2.44% (95% CI, 1.88–3.16%) for children without VPA exposure [8]. Children with prenatal VPA exposure showed core ASD symptoms including cognitive and language impairment, neurodevelopmental delay, social difficulties, and behavioral abnormalities [9–12].

VPA-exposed two-dimensional (2D) neural cell cultures and rodent animals have been intensively used to explore molecular underpinnings of ASD. Studies showed that VPA exposure impairs neural development and differentiation [13–19]. Moreover, rodent animals and nonhuman primates that are prenatally exposed to VPA lead to autistic-like behaviors in the offspring [20–26], supporting the causal link between maternal VPA exposure and ASD susceptibility. However, cell compositions in 2D cell cultures are usually simple that could not reflect the complex cellular architectures of the human brain. In addition, brain developmental processes and genetic backgrounds in animal models are different from those in the human brain. Using a model that resembles human brain developmental features will deepen our understanding on VPA’s contribution to ASD risk.

Human brain organoids are self-organized three-dimensional cell aggregates derived from pluripotent stem cells including human induced pluripotent stem cells (hiPSCs) [27]. For its advantages on recapitulation of complex cellular architectures and developmental trajectories of the human brain [27–29], brain organoid has become an attractive model to study neurodevelopmental disorders including ASD [30]. For example, Mariani et al. used ASD patient-derived telencephalic organoids and revealed that a shift towards GABAergic neuron fate caused by FOXG1, a forebrain-specific marker, may be a developmental precursor of ASD [30]. A recent study [31] found that VPA exposure at the early stage (day 18) of cortical organoids derived from one male hiPSC line impairs neurodevelopment. However, this study used only one hiPSC line which ignored the previously reported sex-differential effects of VPA [17, 32–34], leading to a potential misinterpretation of VPA’s effects. On the other hand, cell-type-specific molecular changes, which link to ASD etiology [35], was not explored in the study [31]. Investigating cell-type-specific effects of VPA exposure in brain organoids helps to understand VPA’s functional mechanisms in the human brain.

To get insight into VPA’s contribution to ASD risk, we generated human forebrain organoids (hFOs) from two normal hiPSC lines derived from one male (U1M) and one female (U2F). hFOs aged 42 days were exposed to VPA at 1 mM, a clinically relevant concentration [36], for 3 days. hFOs exposed with or without VPA were randomly selected for bulk organoid RNA sequencing (RNA-seq) and single-cell RNA-seq (scRNA-seq). We found that VPA exposure in hFOs affected the expression of many ASD risk-associated genes and led to the disrupted synaptic transmission which has been implicated in ASD etiology [37]. In summary, this study revealed VPA’s connection to ASD risk under human genetic background and in vivo-like conditions, which is valuable for illustrating the etiology of ASD.

## Materials and methods

### hiPSC culture

Two hiPSC lines respectively derived from one healthy male (U1M, 37 years) and one healthy female (U2F, 28 years) were obtained from CellAPY Technology (Beijing, China). Pluripotency and karyotypes of these two hiPSC lines were confirmed by immunofluorescence and karyotype analysis (Supplementary Fig. 1). hiPSCs were cultured in Matrigel (Corning, 354277) -coated plate and supplemented with mTeSR Plus medium (STEMCELL Technologies, 05825) with the medium changed daily.

### Generation of hFOs

The U1M and U2F hiPSC lines (Passage < 30) were used for generating hFOs based on the published methods [27, 29, 38] with some modifications. On day 0, hiPSCs were dissociated into single cells using Accutase solution (Sigma-Aldrich, A6964). About 1 × 10^4^ cells per well were then plated into the 96-well round-bottom ultra-low attachment plate (Corning, 7007) and fed with 100 μL embryoid body (EB) Formation Medium, containing DMEM/F12 medium (Gibco, 31331093) supplemented with 20% Knockout Serum Replacement (Thermo Fisher Scientific, 10828028), 1x GlutaMAX (Gibco, 35050061), 1x non-essential amino acids (NEAA) (Gibco, 11140050), 1x Penicillin/Streptomycin (Gibco, 15070063), 10 μM SB431542 (Selleck, S1067), 200 nM LDN193189 (Selleck, S2618), and 20 μM Y27632 (Selleck, S1049). On day 3, each well was gently added with 50 μL fresh EB Formation Medium. On day 5, each well of the 96-well plate was added with 150 μL Neural Induction Medium consisting of DMEM/F12, 1x GlutaMAX, 1x NEAA, 1x Penicillin/Streptomycin, 10 μM SB431542, 200 nM LDN193189, 1x N2 Supplement (Gibco, 17502048), 1 μg/ml Heparin (MCE, HY-17567), 1 μM CHIR99021 (Selleck, S2924), and 4 ng/ml human WNT-3A (R&D Systems, 5036-WN-500). On day 7, organoids were embedded with Matrigel and cultured in the six-well ultra-low attachment plate (Corning, 3471) containing Neural Induction Medium. On day 11, organoids were transferred to an orbital shaker (65 rpm) and cultured in Organoid Differentiation Medium, consisting of DMEM:F12/Neurobasal (1:1), 1x GlutaMAX, 1x NEAA, 1x Penicillin/Streptomycin, 1x N2 supplement, 1x B27 Supplement (Gibco, 17504044), and 2.5 μg/ml Insulin (MCE, HY-P0035). From day 30, organoids were cultured in Organoid Maturation Medium consisting of Organoid Differentiation Medium with the addition of 10 μM Forskolin (Selleck, S2449), 0.2 mM Ascorbic Acid (STEMCELL Technologies, 72132), 10 ng/ml BDNF (STEMCELL Technologies, 78005), and 10 ng/ml GDNF (STEMCELL Technologies, 78058). The Organoid Differentiation and Maturation Media were changed every 4–5 days. The generated hFOs were characterized by immunofluorescence as described in our previous study [38].

### VPA exposure in hFOs and bulk organoid RNA-seq

Since VPA at 1 mM, a clinically relevant concentration [36], was reported to exert nominal effects on neuron differentiation with minimal cell toxicity [39], hFOs at day 42 were fed with fresh Organoid Maturation Medium supplemented with or without 1 mM VPA (Selleck, S1168). After 3 days of VPA exposure, whole organoids (*N* = 3) in each group were randomly selected for total RNA extraction using the miRNeasy Mini Kit (Qiagen, 217004). The quality of RNA samples was evaluated using the Agilent 2100 Bioanalyzer system. RNA samples with RNA integrity numbers over 8 were used for rRNA depletion RNA-seq library construction. The constructed libraries were then used for RNA-seq (150 bp, paired-end) on the Illumina NovaSeq 6000 system.

### Differential gene expression (DGE) analysis in bulk organoid RNA-seq data

Raw RNA-seq reads were filtered to get clean reads using FastQC (v0.20.0). The clean reads were aligned to the human genome GRCh38.p13 using STAR (v2.7.6a). Gene expression quantifications were conducted using RSEM (v1.3.3) based on Gencode v32 comprehensive gene annotations. Quality control metrics were evaluated using PicardTools (v1.139) and Samtools. Genes with transcripts per million values of more than 0.1 in at least 80% samples were retained for DGE analysis. The DGE analysis was performed using the limma package in R (v3.7). *P* values were adjusted using the Benjamini–Hochberg method. To annotate the functions of differentially expressed genes (DEGs), we used the online tool WebGestalt2017 [40] to perform Gene Ontology (GO) and Kyoto Encyclopedia of Genes and Genomes (KEGG) pathway enrichment analysis.

### Protein–protein interaction (PPI) analysis

The STRING database (v11.0) (http://www.string-db.org/) was used to construct a PPI network for the VPA-induced DEGs. Active interaction sources included text-mining, experiments, databases, coexpression, neighborhood, gene fusion, and co-occurrence. The minimum required interaction score was 0.7. The PPI network was visualized using Cytoscape (v3.5.1). CytoHubba [41], a Cytoscape plugin, was used to explore hub nodes in the PPI network, based on 12 topological analysis methods including Degree, Clustering Coefficiency, Edge Percolated Component, Maximum Neighborhood Component, Density of Maximum Neighborhood Component, Maximal Clique Centrality and six centralities (Bottleneck, EcCentricity, Closeness, Radiality, Betweenness, and Stress).

### Real-time quantitative PCR (RT-qPCR) analysis

Total RNA was used to generate complementary DNA using HiScript III RT SuperMix for qPCR (+gDNA wiper) (Vazyme, R323-01). ChamQ SYBR qPCR Master Mix (Vazyme, Q711-02) was used for qPCR analysis on the 7500 Fast Real-Time PCR System (Applied Biosystem). *GAPDH* was used as the internal reference gene. Three technical replicates were used in the RT-qPCR analysis. RT-qPCR primers for *PRKCB* (Forward, AGCCCCACGTTTTGTGACC; Reverse, GCTGGGAACATTCATCACGC) and internal control *GAPDH* (Forward, AATCCCATCACCATCTTCCA; Reverse, TGGACTCCACGACGTACTCA) were derived from PrimerBank [42].

### ScRNA-seq and data analysis

For 10x Genomics scRNA-seq, three hFOs in each group were pooled together and dissociated into single cells using Accutase for 10 min at room temperature. Cells were then centrifuged at 300×*g* for 7 min. The supernatant was removed and cell pellets were washed by PBS with 0.04% BSA. Single cells were then collected using 40 µm strainers and used for Trypan blue cell counting. About 2 × 10^4^ cells per pooled sample were loaded for cell capture, reverse-transcription, and library constructions. The constructed libraries were sequenced on Illumina NovaSeq 6000 system (150 bp, paired-end).

The sequencing data were processed (including alignment and quantification) using Cell Ranger (v6.0.0) pipeline (10x Genomics). Filtering criteria “min.cells = 3, min.features = 200” was used to preliminary filter the data. Cells with less than 500 detected genes or more than 7437 detected genes (mean + 3 SD) were further filtered out. Cells with more than 10% reads aligned to mitochondrial genes were also excluded. Considering doublets existing in scRNA-seq, we removed doublets that were detected by either DoubletFinder (v2.0.3) or Scrublet (v0.2.2) algorithm.

For cell clustering analysis, we used “IntegrateData” function in the Seurat package to combine data from four samples (U1-NC, U1-VPA; U2-NC, U2-VPA). Clustree (v0.4.3) was then used to cluster cells based on the Louvain algorithm with multilevel refinement. Resolution value = 1 and 30 principal components were used in the Clustree analysis. A total of 21 cell clusters (cluster 0–20) were identified (Supplementary Fig. 2). Published cell-type-specific marker genes identified from scRNA-seq of human fetal brain samples [43] and some canonical marker genes (Supplementary Table 1**)** were used to annotate the cell clusters. Dotplots and Featureplots of the cell-type-specific marker genes were shown in Supplementary Figs. 3, 4. We also calculated correlations between the cell clusters and those identified in the reference human fetal brain scRNA-seq data [43] (Supplementary Fig. 5) to evaluate the cell cluster annotation.

The Wilcoxon test was used to identify cell-type-specific DEGs (*P*_adjust_ < 0.05) between VPA-exposed and control groups. ClusterProfiler (v4.0.2) was used to perform GO enrichment analysis for the cell-type-specific DEGs.

### Microelectrode array (MEA) assay

We used an MEA assay to assess whether VPA exposure affects synaptic transmission in hFOs. Since matrigel-embedded organoids could not sufficiently contact with the MEA electrodes, we generated non-matrigel-embedded hFOs using the STEMdiff™ Ventral Forebrain Organoid Differentiation Kit (STEMCELL Technologies, 08630) which is based on the methods of Birey et al. [44]. hFOs aged 42 days were seeded into the MEA culture wells. After 7 days of culture, hFOs were treated with or without 1 mM VPA. Electro activities were recorded at 30 min, 24 h, and 48 h post VPA exposure using the MEA system (Axion Biosystems). Each recording duration was 5 min. The software Axis Navigator 1.5 was used to analyze the MEA data.

### Statistical analysis

Data were analyzed with GraphPad Prism 7.0 and presented as Mean ± SEM. A two-tailed *t*-test was used to assess the differences between the two groups. The hypergeometric test was used to assess the enrichment among VPA-induced DEGs and ASD-associated gene sets. The limma package was used to perform DGE analysis in bulk organoid RNA-seq data. The Wilcoxon test was used to identify cell-type-specific DEGs in the scRNA-seq data. *P* values in the DGE analysis were adjusted for multiple testing using the Benjamini–Hochberg method. For all statistical analyses, a *P* value less than 0.05 is considered statistically significant.

## Results

### VPA-induced DEGs in hFOs are enriched in neuronal functions and pathways

**Fig. 1:**
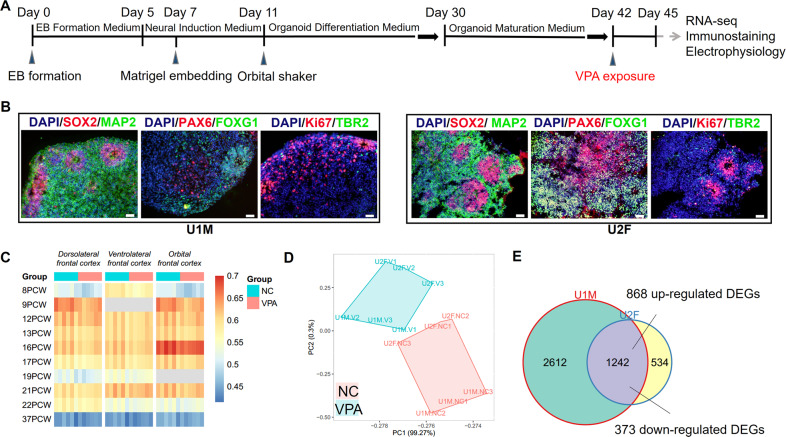
Identification of genes affected by VPA exposure in hFOs. **A** Workflow of this study. **B** Immunofluorescence characterization for hFOs. Ventricular zone marker, SOX2 and ki67; intermediate zone marker, TBR2; cortical plate markers, MAP2; forebrain-specific markers, FOXG1 and PAX6. Scale bar, 50 μm. **C** Transcriptome correlation analysis between hFOs (day 45) and human fetal brains. PCW post-conception weeks. The color bar indicates the correlation coefficient. **D** Principal component analysis for RNA-seq data from VPA-exposed and control hFOs. **E** Overlap between VPA-induced DEGs identified in U1M and U2F hFOs. Among the 1242 overlapped genes, the expression change directions of *COMT* in U1M and U2F were not consistent. The gene *COMT* was not considered as a VPA-induced DEG, the remaining 1241 shared VPA-induced DEGs between U1M and U2F hFOs.

To investigate VPA’s effects on ASD risk in humans, we generated hFOs from two normal hiPSC lines (U1M, male; U2F, female) (Fig. 1A, B). On day 42 of differentiation, hFOs were exposed to 1 mM VPA for 3 days and then randomly selected for RNA-seq to identify genes affected by VPA exposure. Transcriptome correlation analysis between RNA-seq data from hFOs and human fetal brains [45] showed that hFOs aged 45 days mapped most closely to human fetal brains at 9 to 16 post-conception weeks (Fig. 1C). Principal component analysis revealed distinct clusters between VPA-exposed and control hFOs (Fig. 1D). DGE analysis between the VPA-exposed and control hFOs identified 3854 and 1776 DEGs [false discovery rate (FDR) <0.05] in U1M and U2F, respectively (Fig. 1E and Supplementary Table 2). A total of 1242 DEGs overlapped between U1M and U2F, of which 1241 DEGs except *COMT* shared the same expression change directions (Supplementary Table 2) and were considered as putative genes affected by VPA. Among 1241 shared DEGs, 868 genes were upregulated and 373 genes were downregulated in VPA-exposed hFOs.

We next used WebGestalt [46] to annotate biological functions and pathways that 1241 shared DEGs involved in. GO enrichment analysis showed that 868 DEGs upregulated in VPA-exposed hFOs were associated with regulation of transmembrane transport (FDR = 7.30E-05), and multiple neuronal functions, including chemical synaptic transmission (FDR = 7.30E-05), regulation of neurotransmitter levels (FDR = 1.28E-04), as well as potassium ion transport (FDR = 3.80E-03) (Supplementary Fig. 6). KEGG pathway analysis implicated the 868 upregulated DEGs in the oxytocin signaling pathway, dopaminergic, cholinergic, and GABAergic synapses, as well as the calcium signaling pathway (FDR < 4.4E-03) (Supplementary Fig. 6). For the 373 downregulated DEGs in VPA-exposed hFOs, GO, and KEGG pathway analysis revealed their significant enrichment in extracellular structure organization (FDR = 7.25E-04), gliogenesis (FDR = 3.34E-03), peripheral nervous system development (FDR = 7.70E-03), as well as focal adhesion (FDR = 5.62E-03), and ECM-receptor interaction pathways (FDR = 1.78E-02) (Supplementary Fig. 6).

### VPA-induced DEGs in hFOs are enriched with ASD risk-associated genes

**Fig. 2:**
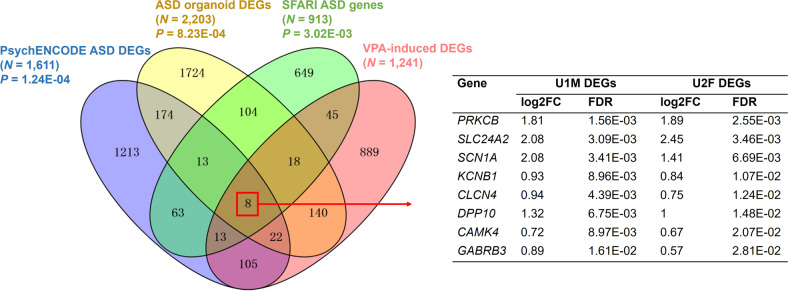
Enrichment of 1241 shared VPA-induced DEGs with ASD risk-associated gene sets. Veen Diagram shows the overlap among VPA-induced DEGs and three sets of ASD-associated genes. The hypergeometric test was used to assess the enrichment between VPA-induced DEGs and ASD-associated gene sets. *P* values of the hypergeometric tests are indicated. Eight DEGs shared in all the gene sets are shown. FC fold change, FDR false discovery rate.

To explore the connection between VPA exposure and ASD risk, we evaluated the enrichment of 1241 VPA-induced DEGs with three sets of ASD risk-associated genes (Supplementary Table 2). The first ASD gene set were 1611 DEGs identified from the PsychENCODE postmortem brain transcriptome data of 51 ASD patients and 936 controls [47]. The second ASD gene set were 2203 DEGs between organoids (day 31) derived from four ASD patients and their respective unaffected fathers from the Mariani et al. study [30]. The third ASD gene set were 913 ASD risk genes from the SFARI database (https://gene.sfari.org/). We found that VPA-induced DEGs were significantly overlapped with genes dysregulated in PsychENCODE ASD brains (Overlapped genes = 148, *P* = 1.24E-04) and ASD patient-derived organoids (Overlapped genes = 188, *P* = 8.23E-04) (Fig. 2). The VPA-induced DEGs were also enriched with ASD risk genes from the SFARI database (Overlapped genes = 84, *P* = 3.02E-03) (Fig. 2). Notably, eight DEGs including *CAMK4*, *CLCN4*, *DPP10*, *GABRB3*, *KCNB1*, *PRKCB*, *SCN1A*, and *SLC24A2*, which are known to be associated with several ion (calcium, sodium, potassium, chloride) channels, were shared in all the three ASD gene sets (Fig. 2).

We also investigated enrichment of the 1241 VPA-induced DEGs with genes in the ASD risk-associated gene coexpression modules identified from the PsychENCODE brain transcriptome data [47]. We found that 138 of 1241 DEGs were significantly (*P* = 4.23E-02) overlapped with genes in ASD risk-associated module 3 (Module genes = 1747), which was enriched in multiple neuronal functions, including gliogenesis (FDR = 3.44E-08) and neural tube development (FDR = 3.35E-05). We also found that 13 of 1241 DEGs were enriched with (*P* = 4.65E-03) genes in ASD risk-associated module 23 (Module genes = 93), which was associated with neuronal functions, including neuropeptide signaling pathway (FDR = 2.12E-04), neuron migration (FDR = 8.40E-04), and modulation of synaptic transmission (FDR = 1.30E-03).

### VPA-induced DEGs in hFOs are clustered in a PPI network

**Fig. 3:**
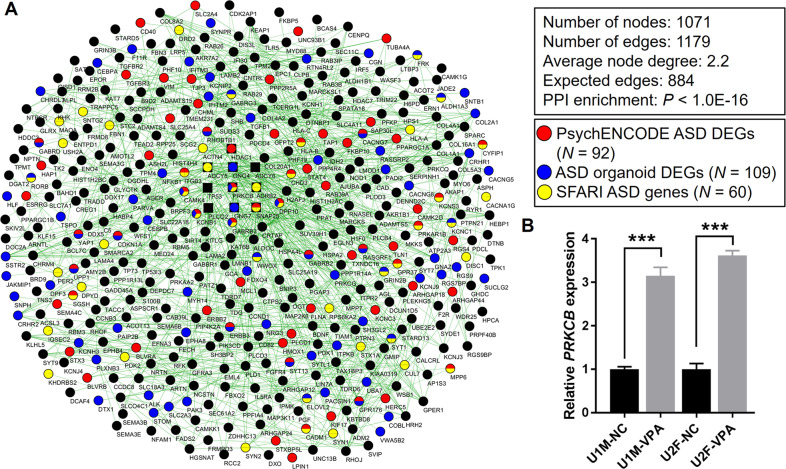
PPI network for 1241 shared VPA-induced DEGs between U1M and U2F hFOs. **A** The colors of nodes indicate different ASD-associated gene sets. The number of ASD-associated genes in the PPI network is also indicated. The top ten hub nodes ranked by connectivity degree in the PPI network are shown in the rectangle. Nodes that don’t connect with the hub nodes are not shown. The width of edges represents the PPI confidence score. **B** RT-qPCR was used to test *PRKCB* expression in the VPA-exposed and control hFOs. *PRKCB* expression in the control group was normalized to 1. Data were shown as Mean ± SEM (organoids *N* = 7 from two batches). Two-tailed *t*-test, ***P < 0.001.

We further investigated the functions of VPA-induced DEGs using PPI information from the STRING database [48]. We found that 571 of 1241 shared DEGs were significantly clustered into a high-confidence PPI network (interaction score >0.7, *P* < 1.0E-16) with a majority of the overlapped ASD risk genes included (Fig. 3A and Supplementary Table 3). Functional annotation revealed enrichment of the PPI network in regulation of ion transport (FDR = 7.3E-04) such as potassium ion transmembrane transport (FDR = 2.01E-02), nervous system development (FDR = 4.8E-03), and positive regulation of synaptic transmission (FDR = 4.8E-03). The PPI network was also enriched in Reactome pathways including neuronal system (FDR = 1.04E-06), transmission across chemical synapses (FDR = 2.34E-05), potassium channels (FDR = 2.77E-02), and GABA receptor activation (FDR = 2.9E-02) (Supplementary Table 3). Using CytoHubba [41], a method used to explore hub nodes in biological networks, we identified proteins, including TP53, GNG4, GNG7, SNAP25, ADCY6, PRKCB, ADCY5, HDAC1, ADRB2, and PLCG2, as the top ten most connected nodes ranked by connectivity degree in the PPI network. It was worth noting that 7 of 12 algorithms available in CytoHubba identified PRKCB, a beta type of protein kinase C, as one of the top ten hub nodes in the PPI network (Supplementary Table 3).

Considering that PRKCB is a hub node in the PPI network and is a shared gene among the aforementioned three ASD gene sets, we prioritized *PRKCB* as a potential hub gene that mediates VPA’s effects on ASD risk. We used RT-qPCR analysis and validated the upregulation of *PRKCB* expression in response to VPA exposure in hFOs (Fig. 3B).

### ScRNA-seq identifies cell-type-specific effects of VPA exposure in hFOs

To get insight into VPA’ effects in the human brain that consists of multiple cell types, we performed scRNA-seq to identify cell-type-specific effects of VPA exposure in hFOs. hFOs aged 50 days that were exposed with or without VPA were used for 10x Genomics scRNA-seq. A total of 3199 and 7620 cells were sequenced in the VPA-exposed (U1-VPA, 1043; U2-VPA, 2156) and control samples (U1-NC, 5727; U2-NC, 1893), with an average of 1844 and 3417 genes per cell detected in the VPA-exposed and control group, respectively. Clustering analysis identified 21 cell clusters (cluster 0–20) for the total 10,819 cells (Supplementary Fig. 2). DGE analysis between any individual cluster and all the others identified sets of marker genes for each cell cluster (Supplementary Table 4).

**Fig. 4:**
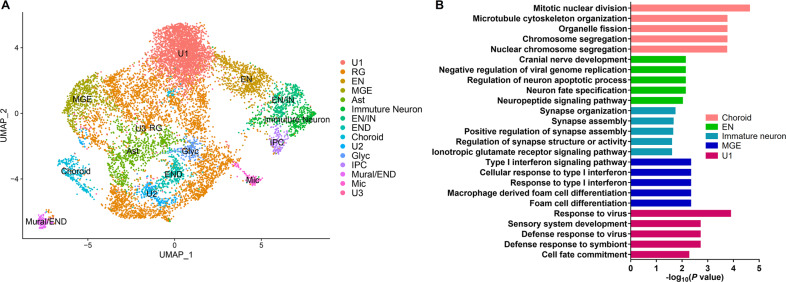
scRNA-seq analysis reveals cell-type-specific effects of VPA exposure in hFOs. **A** UMAP scatterplot for a total of 10,819 cells derived from U1M and U2F hFOs. Ast astrocyte, Choroid choroid plexus, EN excitatory neuron, END endothelia, Glyc glycolysis, IN inhibitory neuron, IPC intermediate progenitor cells, MGE medial ganglionic eminence, Mic microglia, Mural mural cell, RG radial glia, U1–U3 unknown cell clusters 1–3. **B** GO enrichment analysis for DEGs identified in the annotated cell types of hFOs. The top five GO terms are shown.

Using published cell-type-specific markers from scRNA-seq of human fetal brain samples [43] and canonical cell-type markers (Supplementary Table 1), ten major cell types including astrocyte (Ast), choroid plexus (Choroid), endothelia (END), intermediate progenitor cells (IPC), medial ganglionic eminence (MGE), radial glia (RG), immature neuron, excitatory and inhibitory neuron (EN and IN), and microglia-like cells were identified (Fig. 4A). RG cells and neurons (7% EN, 3% immature neuron, 3% EN/IN) accounted for 32 and 13% of the total cells, respectively (Supplementary Fig. 7). DGE analysis showed that VPA affected the expression of genes in the choroid, EN, immature neuron, MGE cells, and unknown cell cluster 1 (Supplementary Table 4). GO enrichment analysis identified cell-type-specific functions for the VPA-induced DEGs (Fig. 4B and Supplementary Table 4). For example, DEGs in choroids were enriched with mitotic nuclear division, multiple neural functions including neuron death, differentiation, and migration. DEGs in EN and immature neurons were also enriched with multiple neural functions such as neural development, synapse organization, and synaptic transmission. Interestingly, VPA-induced DEGs in MGE and the unknown cluster 1 were related to type I interferon and virus response, implicating that VPA exposure in hFOs may trigger an immune response (Fig. 4B and Supplementary Table 4).

### VPA affects synaptic transmission in hFOs

**Fig. 5:**
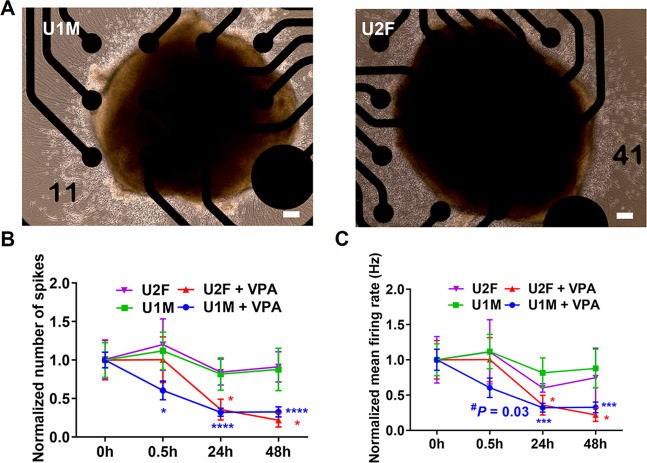
VPA affects synaptic transmission in hFOs. **A** Bright fields for hFOs cultured on the MEA plate. Scale bar, 100 μm. **B**, **C** Number of spikes (**B**) and mean neuron firing rate (**C**) post VPA exposure. Data were shown as Mean ± SEM (averaged organoids *N* = 7 from two batches). Number of spikes and mean neuron firing rate in the control groups (0 h) were normalized to 1. The student’s *t*-test was used to assess differences between the control (0 h) and the other VPA-exposed group (30 min, 24 h, 48 h). ^#^*P* < 0.05, one-tailed *t*-test. *P < 0.05, ***P < 0.001, ****P < 0.0001, two-tailed *t*-test.

Since VPA-induced DEGs were found to be associated with synaptic transmission, calcium, and potassium signaling pathways, we used an MEA system to investigate whether VPA affects the electrophysiological properties of hFOs. hFOs (day 42) from both U1M and U2F cell lines were seeded into a 24-well MEA plate. After 7 days of culture in the MEA plate (Fig. 5A), hFOs were exposed with or without 1 mM VPA for 30 min, 24 h, and 48 h. For hFOs without VPA exposure, the number of spikes and mean neuron firing rate didn’t show notable changes within the 48-h window, while those hFOs from both U1M and U2F that were exposed to VPA showed significantly (Two-tailed *t*-test, *P* < 0.05) decreased the number of spikes and mean neuron firing rate at 24 and 48 h post VPA exposure (Fig. 5B, C). These results suggest that VPA exposure in hFOs disrupts synaptic transmission, which is thought to be a major pathological mechanism for ASD [37].

## Discussion

Prenatal VPA exposure as an environmental factor reported to be associated with increased ASD risk, the functional mechanisms of which in the human brain are not clear. Our understanding about VPA’s effects is largely based on 2D cell cultures and animal models which could not recapitulate complex cellular architectures and developmental trajectories of the human brain, investigations under human genetic background and in vivo-like physiological conditions are required. In this study, we used hFOs, a model that recapitulates key features of human brain development, to identify genes and pathways affected by VPA. We identified 1241 shared VPA-induced DEGs between U1M and U2F hFOs, which were enriched in neural development, synaptic transmission, and various signaling pathways including the oxytocin signaling pathway. It’s worth noting that the oxytocin signaling pathway has been implicated in ASD and is considered as a potential therapeutic target for ASD [49, 50], supporting the connection between VPA exposure and the etiology of ASD. Interestingly, 734 of the 1241 VPA-induced DEGs significantly (*P* = 3.96E-39) overlapped with those (*N* = 7418) identified in VPA-exposed cerebral organoids from a recent study [31] (Supplementary Table 2), suggesting the robust findings of our study. To be noted, the VPA-induced DEGs in our study significantly overlapped with SFARI ASD risk genes which were supported by genetic evidence, suggesting that VPA and ASD-associated genetic variations converge into molecular functions and pathways contributing to ASD risk.

To get further insight into VPA’s potential action mechanisms in the human brain, we performed scRNA-seq to identify cell-type-specific effects of VPA exposure in hFOs. scRNA-seq identified diverse brain cell types annotated in hFOs. DGE analysis in the scRNA-seq data showed that VPA exposure affected gene expression in the choroid, EN, immature neuron, MGE cells, and unknown cell cluster 1 which accounted for 27% of the total cells (Supplementary Fig. 7). GO enrichment analysis further identified cell-type-specific effects of VPA exposure in hFOs, implying that VPA may have distinct functional impacts in different cell types in the human brain. Intriguingly, scRNA-seq appeared to identify microglia-like cells (cluster 18) in hFOs since cluster marker genes for cluster 18, including immediate early genes *JUN*, *JUNB*, *IER2*, and *FOS*, were significantly overlapped with those in microglia clusters identified from human fetal brain scRNA-seq studies [51, 52]. However, expression of canonical microglia marker genes including *IBA1*, *CD11b*, and *PTPRC* were not detected in cluster 18, implying that cells in cluster 18 were not mature microglia.

In addition to gene expression alterations, VPA exposure in hFOs disrupted synaptic transmission, which is thought to be a major ASD pathological mechanism [37]. Interestingly, we found that VPA seems to have sex-differential effects on synaptic transmission, since the number of spikes and mean neuron firing rate deceased in U1M male but not in U2F female hFOs at 30 min post VPA exposure (Fig. 5B, C). Regarding sex difference, we found that *COMT*, a gene linked to sex differences in brain function and predisposition to psychiatric disorders [53], was upregulated in U1M but downregulated in U2F hFOs post VPA exposure, suggesting that *COMT* may mediate the sex-differential effects of VPA exposure. Additionally, we found that expression of *HDAC7*, a member of histone deacetylase family, was downregulated upon VPA exposure in both U1M and U2F hFOs, however, VPA-induced downregulated DEGs in U1M male but not U2F female hFOs were enriched for histone acetylation (Supplementary Table 5), a canonical VPA action pathway [54]. These results suggest that VPA exposure in hFOs may have sex-differential effects.

This study reveals the effects of VPA exposure in hFOs and provides evidence for illuminating VPA’s potential functional mechanisms in the human brain, some limitations also exist. First, hFOs aged 45 days in this study mimic early stages (up to 16 post-conception weeks) of the human fetal brain, how VPA functions in late brain developmental stages are not clear. Second, though scRNA-seq detected IN markers specifically expressed in cluster 10, EN markers were also highly specifically expressed in this cluster (Supplementary Fig. 3), suggesting that hFOs were not mature enough to contain abundant INs. The effects of VPA exposure on INs remained undiscovered in this study. Third, though we highlighted several hub genes (e.g., *PRKCB*) that may mediate the effects of VPA, how they connect VPA to ASD risk are not clear. In addition, which genes or pathways may mediate VPA’s effects on synaptic transmission are also unclear, further investigations are needed. Last, the number of hiPSC lines used in this study is limited, more cell lines could be used in the future study to investigate potential sex-differential effects of VPA exposure.

In summary, our study investigated VPA’s potential functional mechanisms under human genetic background and in vivo-like conditions based on hFOs. We highlight that VPA exposure may contribute to ASD risk in humans by affecting biological functions and the expression of genes related to ASD.

## Supplementary information

Supplementary Information

Supplementary Table 1

Supplementary Table 2

Supplementary Table 3

Supplementary Table 4

Supplementary Table 5

## Data Availability

Raw data of bulk organoids RNA-seq and scRNA-seq are deposited in the Gene Expression Omnibus (Accession numbers: GSE187006 and GSE186814). Raw MEA data are available on request.
