# Supplementary material for: Human forebrain organoids reveal connections between valproic acid exposure and autism risk
Source: Transl Psychiatry. 2022 Mar 29;12:130. doi: 10.1038/s41398-022-01898-x (PMC8964691; doi:10.1038/s41398-022-01898-x)
Supplement: Supplementary file 1 — Supplementary Information [file 41398_2022_1898_MOESM1_ESM.pdf]

Supplementary Figures

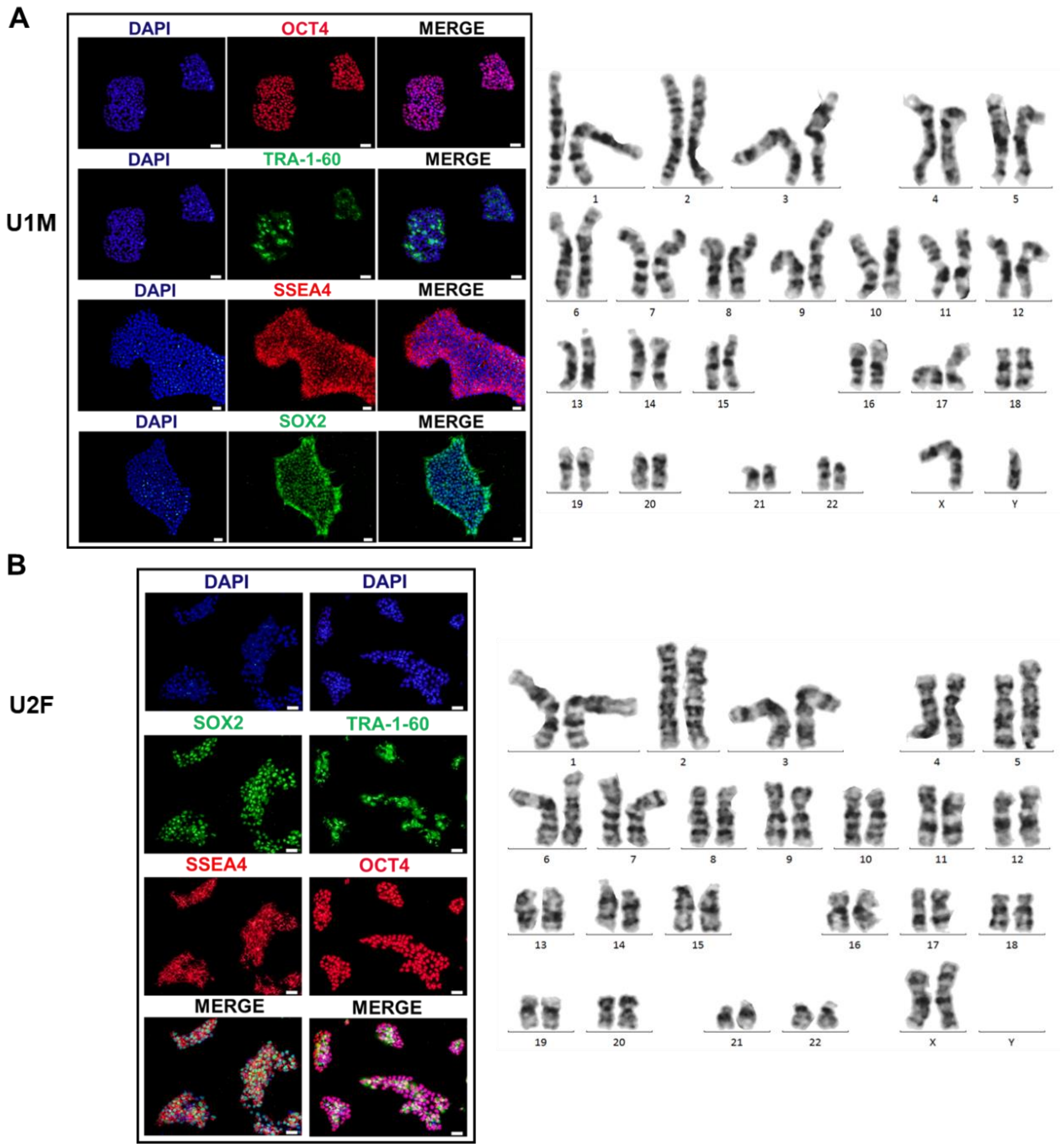

Supplementary Fig. 1. Pluripotency and karyotype analysis of U1M and U2F hiPSC lines. Immunofluorescence staining and karyotype analysis were used to characterize the hiPSC lines U1M (A) and U2F (B). The karyotype of U1M is derived from our previous study [1]. Cell surface antigens for pluripotent stem cells, SSEA-4 and TRA-1-60; pluripotency markers, OCT4 and SOX2. Scale bar, 50 µm.

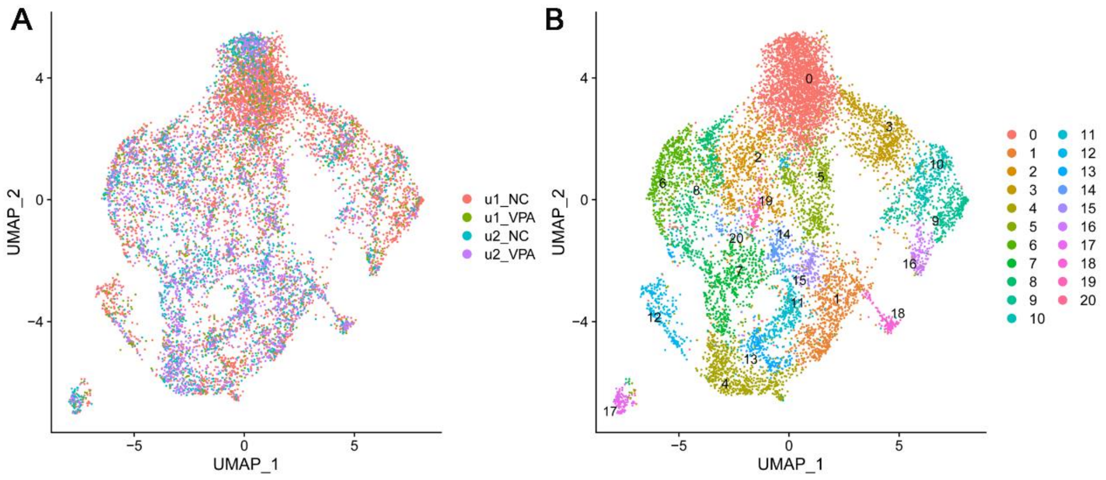

Supplementary Fig. 2. UMAP scatterplot of 10,819 cells from U1M and U2F hFOs. Clustree (v0.4.3) was used to cluster cells based on the Louvain algorithm with multilevel refinement. Cells were labeled with names of samples (A) or IDs of clusters (B). A total of 21 clusters were identified.

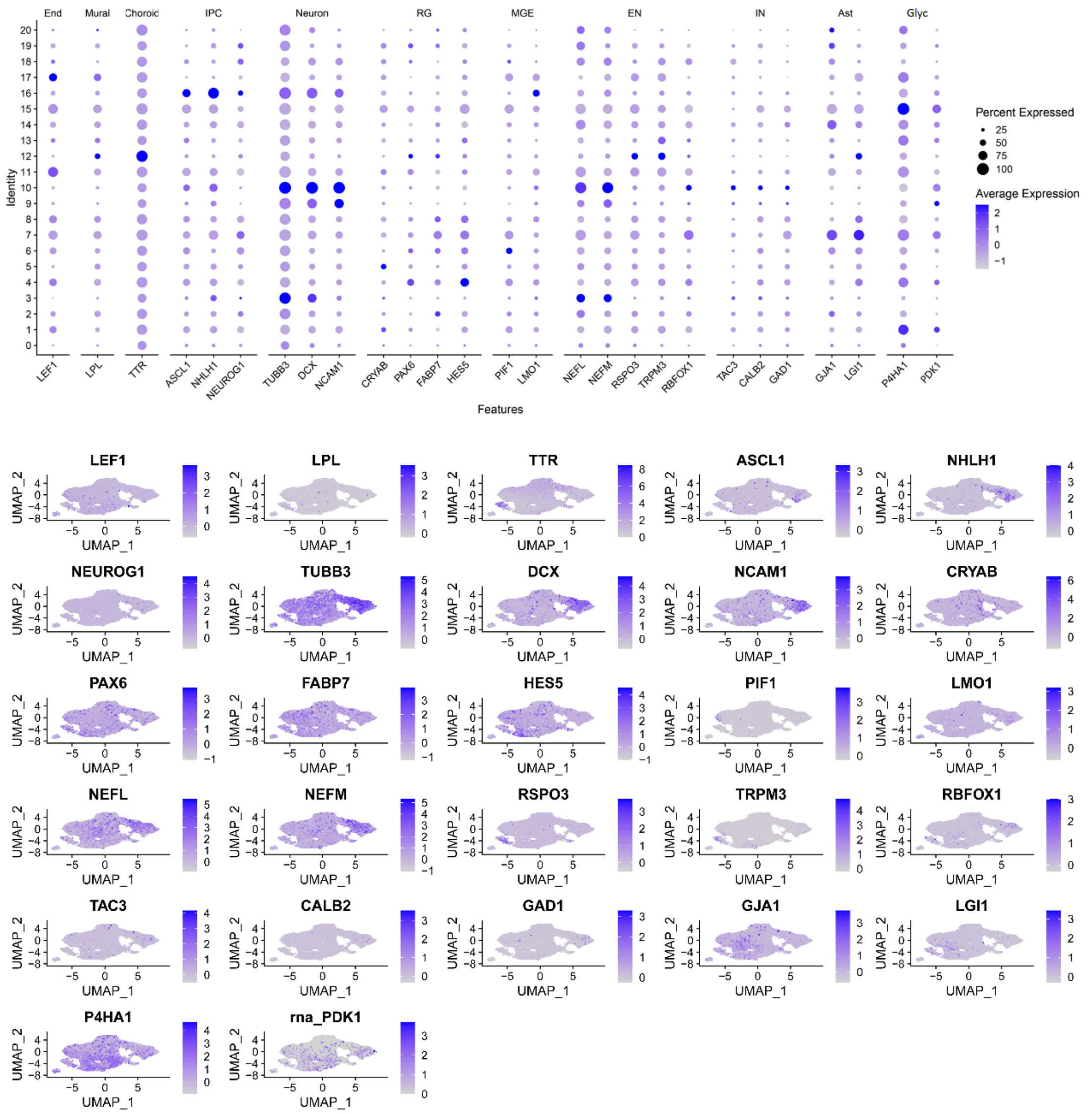

Supplementary Fig. 3. Dotplots and Featureplots of cell type marker genes in scRNA-seq data. Astrocyte (*GJA1*, *LGI1*); Choroid plexus (*TTR*); Excitatory neuron (*NEFL*, *NEFM*, *RSPO3*, *TRPM3*, *RBFOX1*); Endothelia (*LEF1*); Glycolysis (*PDK1*, *P4HA1*); Inhibitory neuron (*TAC3*, *CALB2*, *GAD1*); Intermediate progenitor cells (*ASCL1*, *NHLH1*, *NEUROG1*); Medial ganglionic eminence (*PIF1*, *LMO1*); Mural cell (*LPL*); Radial glia (*CRYAB*, *PAX6*, *FABP7*, *HES5*). *P4HA1* was identified as a glycolysis marker from published studies [2,3]. *TUBB3*, *DCX*, and *NCAM1* are canonical markers for immature neurons. *RBFOX1* and *GAD1* are canonical markers for excitatory and inhibitory neuron, respectively.

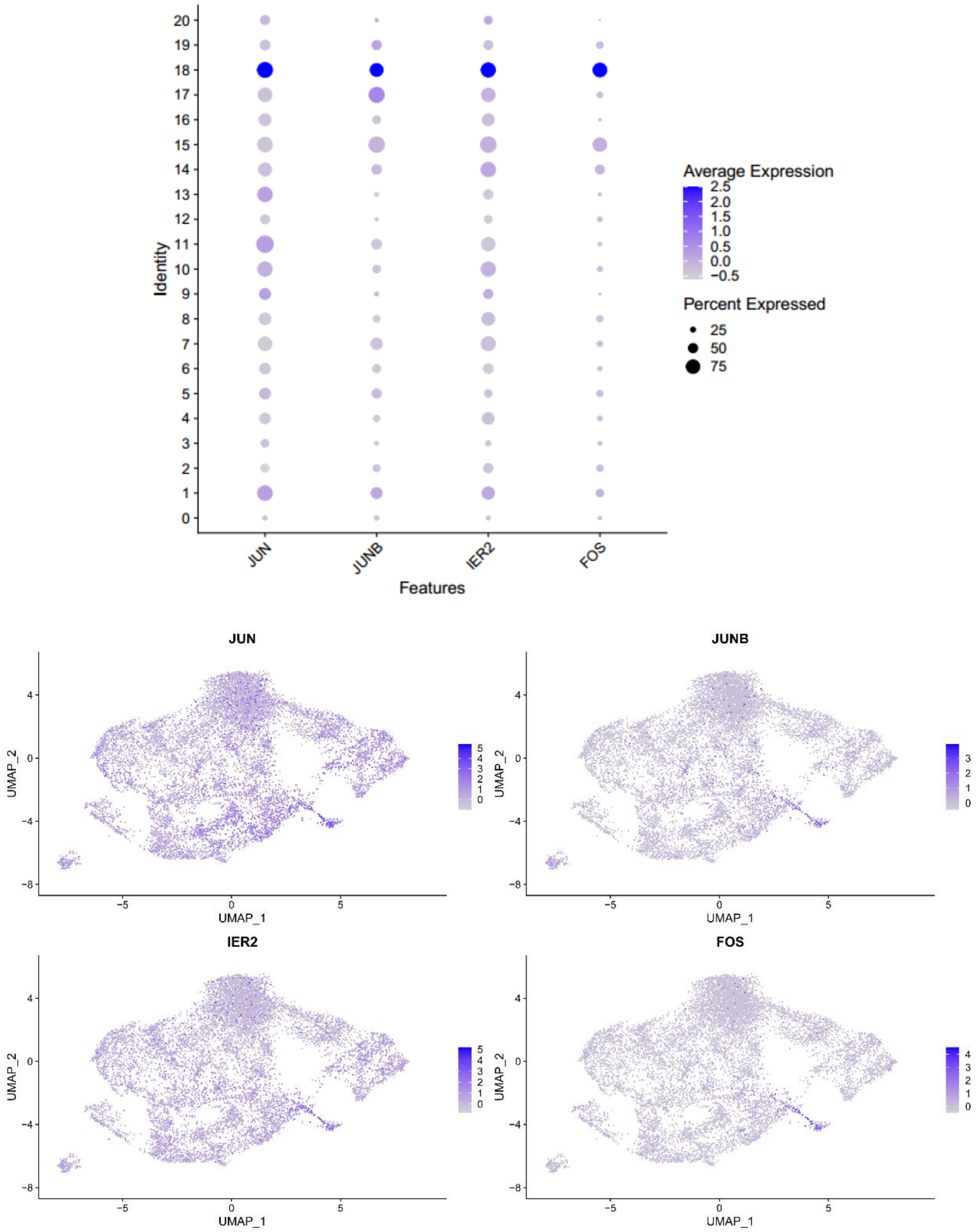

Supplementary Fig. 4. Dotplots and Featureplots of microglia marker genes in scRNA-seq data. Microglia marker genes, *JUN*, *JUNB*, *IER2*, and *FOS*. These four genes were identified as microglia cluster markers from scRNA-seq studies of mouse [4] and human fetal brain samples [5,6].

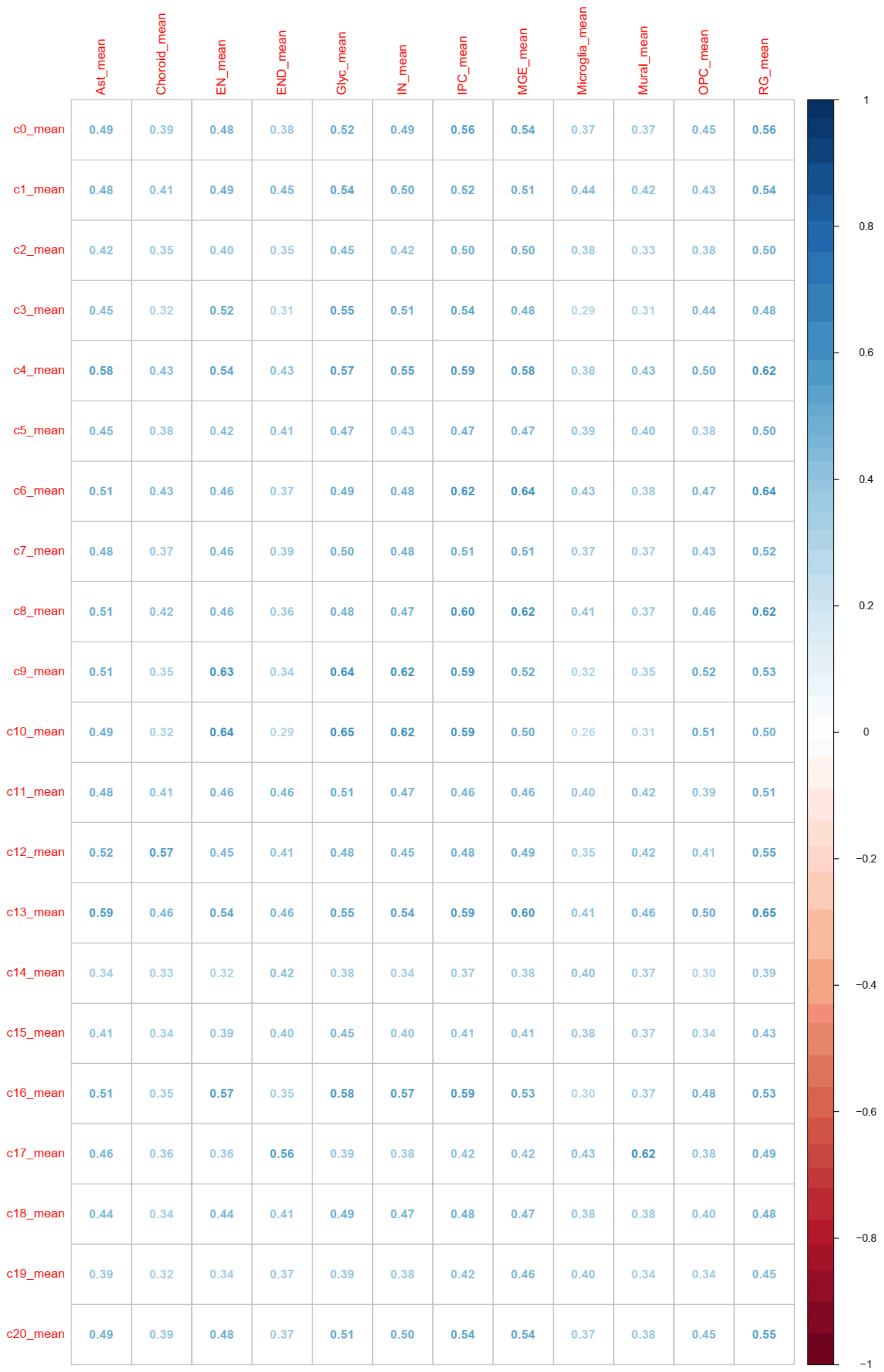

	Ast_mean	Choroid_mean	EN_mean	END_mean	Glyc_mean	IN_mean	IPC_mean	MGE_mean	Microglia_mean	Mural_mean	OPC_mean	RG_mean
c0_mean	0.49	0.39	0.48	0.38	0.52	0.49	0.56	0.54	0.37	0.37	0.45	0.56
c1_mean	0.48	0.41	0.49	0.45	0.54	0.50	0.52	0.51	0.44	0.42	0.43	0.54
c2_mean	0.42	0.35	0.40	0.35	0.45	0.42	0.50	0.50	0.38	0.33	0.38	0.50
c3_mean	0.45	0.32	0.52	0.31	0.55	0.51	0.54	0.48	0.29	0.31	0.44	0.48
c4_mean	0.58	0.43	0.54	0.43	0.57	0.55	0.59	0.58	0.38	0.43	0.50	0.62
c5_mean	0.45	0.38	0.42	0.41	0.47	0.43	0.47	0.47	0.39	0.40	0.38	0.50
c6_mean	0.51	0.43	0.46	0.37	0.49	0.48	0.62	0.64	0.43	0.38	0.47	0.64
c7_mean	0.48	0.37	0.46	0.39	0.50	0.48	0.51	0.51	0.37	0.37	0.43	0.52
c8_mean	0.51	0.42	0.46	0.36	0.48	0.47	0.60	0.62	0.41	0.37	0.46	0.62
c9_mean	0.51	0.35	0.63	0.34	0.64	0.62	0.59	0.52	0.32	0.35	0.52	0.53
c10_mean	0.49	0.32	0.64	0.29	0.65	0.62	0.59	0.50	0.26	0.31	0.51	0.50
c11_mean	0.48	0.41	0.46	0.46	0.51	0.47	0.46	0.46	0.40	0.42	0.39	0.51
c12_mean	0.52	0.57	0.45	0.41	0.48	0.45	0.48	0.49	0.35	0.42	0.41	0.55
c13_mean	0.59	0.46	0.54	0.46	0.55	0.54	0.59	0.60	0.41	0.46	0.50	0.65
c14_mean	0.34	0.33	0.32	0.42	0.38	0.34	0.37	0.38	0.40	0.37	0.30	0.39
c15_mean	0.41	0.34	0.39	0.40	0.45	0.40	0.41	0.41	0.38	0.37	0.34	0.43
c16_mean	0.51	0.35	0.57	0.35	0.58	0.57	0.59	0.53	0.30	0.37	0.48	0.53
c17_mean	0.46	0.36	0.36	0.56	0.39	0.38	0.42	0.42	0.43	0.62	0.38	0.49
c18_mean	0.44	0.34	0.44	0.41	0.49	0.47	0.48	0.47	0.38	0.38	0.40	0.48
c19_mean	0.39	0.32	0.34	0.37	0.39	0.38	0.42	0.46	0.40	0.34	0.34	0.45
c20_mean	0.49	0.39	0.48	0.37	0.51	0.50	0.54	0.54	0.37	0.38	0.45	0.55

Supplementary Fig. 5. Correlations among cell clusters and those identified in the reference human fetal brain scRNA-seq data. The reference scRNA-seq data were from Nowakowski et al. study [7]. The color bar indicates the correlation coefficients.

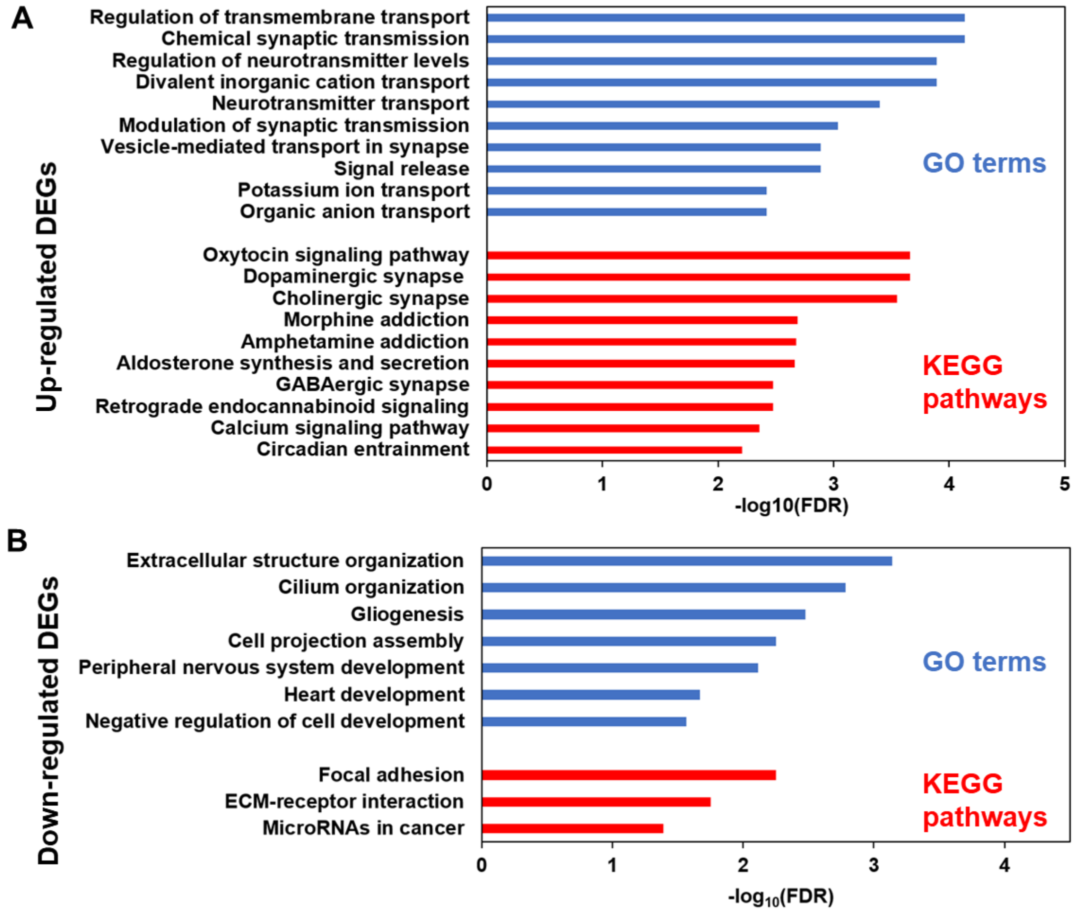

Supplementary Fig. 6. Functional annotation for 1,241 shared VPA-induced DEGs in both U1M and U2F hFOs. GO and KEGG pathway analysis for up-regulated (A) and down-regulated (B) VPA-induced DEGs. The blue and red bars indicate GO terms and KEGG pathways, respectively. Top 10 GO or KEGG terms are shown. FDR, false discovery rate.

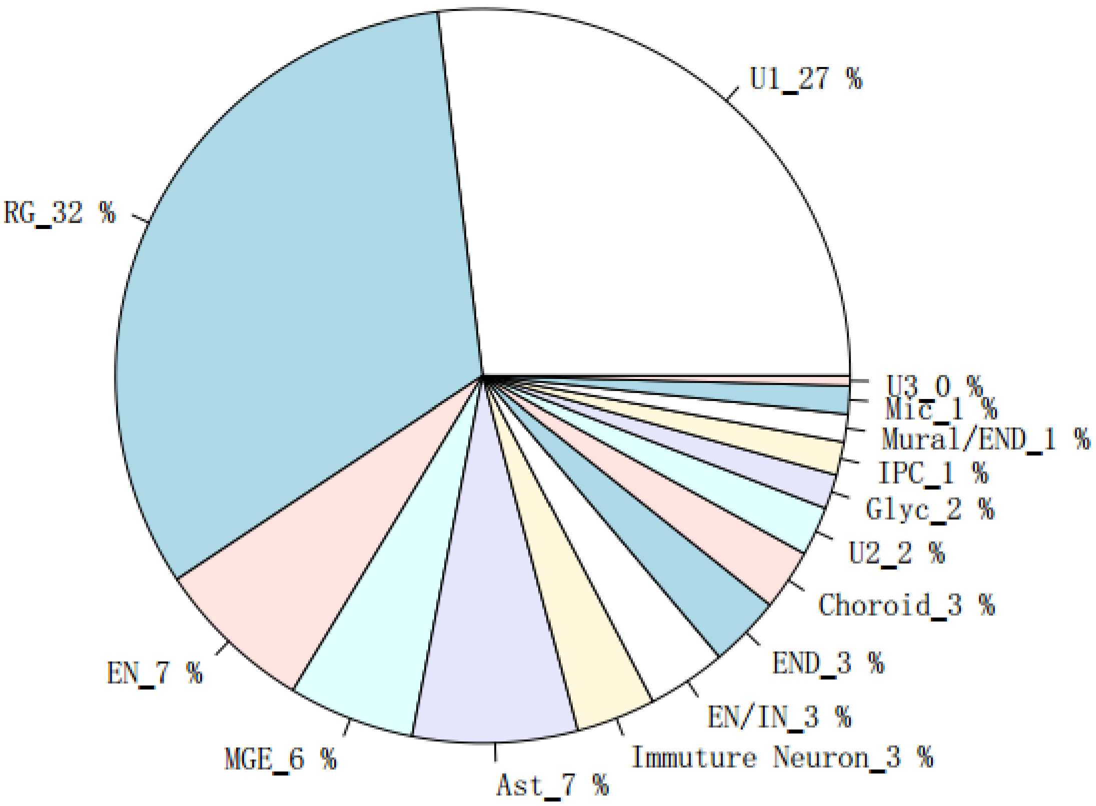

Supplementary Fig. 7. Cell proportion of each cell cluster in the total of 10,819 cells. Ast, astrocyte; Choroid, choroid plexus; EN, excitatory neuron; END, endothelia; Glyc, Glycolysis; IN, inhibitory neuron; IPC, intermediate progenitor cells; MGE, medial ganglionic eminence; Mic, microglia; Mural, mural cell; RG, radial glia; U1-U3, unknown cell clusters 1-3.

References

1 Meng Q, Wang L, Dai R, Wang J, Ren Z, Liu S, et al. Integrative analyses prioritize GNL3 as a risk gene for bipolar disorder. Mol Psychiatry. 2020;25:2672-2684.

2 Cao XP, Cao Y, Li WJ, Zhang HH, Zhu ZM. P4HA1/HIF1alpha feedback loop drives the glycolytic and malignant phenotypes of pancreatic cancer. Biochem Biophys Res Commun. 2019;516:606-612.

3 Wei J, Huang K, Chen Z, Hu M, Bai Y, Lin S, et al. Characterization of Glycolysis-Associated Molecules in the Tumor Microenvironment Revealed by Pan-Cancer Tissues and Lung Cancer Single Cell Data. Cancers (Basel). 2020;12.

4 Ochocka N, Segit P, Walentynowicz KA, Wojnicki K, Cyranowski S, Swatler J, et al. Single-cell RNA sequencing reveals functional heterogeneity of glioma-associated brain macrophages. Nat Commun. 2021;12:1151.

5 Zhong S, Zhang S, Fan X, Wu Q, Yan L, Dong J, et al. A single-cell RNA-seq survey of the developmental landscape of the human prefrontal cortex. Nature. 2018;555:524-528.

6 Fan X, Dong J, Zhong S, Wei Y, Wu Q, Yan L, et al. Spatial transcriptomic survey of human embryonic cerebral cortex by single-cell RNA-seq analysis. Cell Res. 2018;28:730-745.

7 Nowakowski TJ, Bhaduri A, Pollen AA, Alvarado B, Mostajo-Radji MA, Di Lullo E, et al. Spatiotemporal gene expression trajectories reveal developmental hierarchies of the human cortex. Science. 2017;358:1318-1323.
